# Assessing willingness to accept a compensation for evicted land in peri-urban areas: A contingent valuation method

**DOI:** 10.1016/j.heliyon.2024.e40209

**Published:** 2024-11-09

**Authors:** Tadele Alamneh, Melkamu Mada, Tora Abebe

**Affiliations:** Department of Economics, College of Business and Economics, Arba Minch University, Arba Minch, Ethiopia

**Keywords:** Compensation, Willingness to accept, Contingent valuation method

## Abstract

Urban expansion in developing countries like Ethiopia is causing political turmoil in peri-urban areas due to inadequate compensation for evicted farmers because of the absence of a land market, which is prohibited by Ethiopia's land policy. This paper applies the contingent valuation method to measure the compensation value considered acceptable to those who are evicted from their farm land. The west Gojjam and Awi zones of Amhara region, Ethiopia are chosen for the case study based on 393 completed questionnaires. It has been demonstrated that a willingness to accept compensation is essential for maintaining sustainable livelihoods. The study applied single-bounded contingent valuation and double-bounded contingent valuation methods to estimate the mean willingness to accept compensation value. Based on the finding, double-bounded contingent valuation could be more optimal for the welfare of the evictees in detecting and correcting respondent's bias due to its narrow confidence interval. Pilot survey was employed to ensure reliability and validity of the methods employed. Therefore, the optimal bid vector for multiplying the average land output preceding three years of eviction is 29.27. Additionally, variables such as initial bid, age, education, and total annual income significantly affect the willingness to accept compensation. The study recommends that federal and local governments should provide adequate compensation offers that prioritize the well-being of peri-urban communities during urban expansion programs, as farmers are losing their most valuable asset. This is crucial for sustainable living and poverty reduction among those who have been evicted.

## 1 Introduction

The urbanization rate in Africa is increasing alarmingly compared to the rest of the world, although it remains the least urbanized region globally. By 2021, the whole of Africa had only 44.3 % of its population living in urban areas [1]. This proportion lags far behind that of the OECD, Latin America and the Caribbean, and the Arab States, which are, respectively, 57 %, 77 %, and 75 %. However, Africa has the fastest-growing urban population in the world, with annual growth rates approaching 4.4 percent. It is predicted that half of Africa's population will live in cities by 2030. Moreover [2], projected that by 2040, there will be one billion people living in cities across Africa, up from 395 million in 2010 [3]. This indicates that urbanization in Arica will increase alarmingly demanding more agricultural land to the urbanities affecting the peri-urban evictees.

Ethiopia is significantly under-urbanized, even by the African standards. The slow pace of urbanization continued until the mid-1930s, after which it accelerated [4]. This fast urbanization was most visible in Ethiopia's northern half, where the majority of large cities are located. As part of northern Ethiopia, the situation of Amhara regional state urbanization is growing faster (6 %) compared to other regions and the national growth rate (4 %) [5] (see Annex Table 1).

The rapid process of urbanization has significant consequences for peri-urban farmers. One of the primary impacts is the conversion of peri-urban agricultural land into urban residential areas. As a result, farmers in these areas are faced with the displacement of their traditional peasant livelihoods, accompanied by the loss of valuable agricultural land [6]. This conversion is driven by the rapid expansion of cities into the periphery, motivated by the need for urban residential settlements and the development of public infrastructure [7,8]. As urban areas continue to grow extensively, the number of displaced farmers increases, leading to a multitude of challenges. The foremost challenge is the limited availability of agricultural opportunities for these farmers. The loss of agricultural land and the diminishing opportunities for farming not only disrupt their way of life but also threaten their economic stability. Without viable alternatives or support systems in place, these farmers may find themselves pushed towards destitution, facing severe economic hardships and uncertainties about their future [7].

To ensure the well-being of affected individuals, compensation for forced acquisition of buildings and land often relies on the agreement between buyers and sellers. In some cases, such as when land is acquired for development project purposes, a replacement cost model may be utilized as a compensation when market value is unclear [9]. However, compensation practices vary across countries, with different approaches. In some nations, the government sets compensation prices, sometimes consulting with affected farmers and in other cases, without their input. For instance, according to the study by Ref. [10], in Zambia, laws mandate adequate compensation for individuals facing eviction due to development projects, funded by Parliament. The Land Acquisition Act guides the compensation process based on market value and principles like reflecting the property's sale price. Uganda involves negotiations between landowners and project stakeholders, with the right to hire valuers. In Kenya, compensation aligns with market values. China's compensation for acquired land includes various fees and subsidies for affected farmers [11]. India uses different methods, like multiplying the market value of the land (by four for rural land and two for urban land) to determine compensation. India also includes interest, allowances, and additional percentages based on the acquisition's nature. Each country's approach reflects its unique land acquisition and compensation framework [12].

Nevertheless, compensation estimates worldwide are influenced by various factors, including the cost of substitute land, necessary capital, location, utility, type of land, and level of development in the area. However, in Ethiopia, the consideration of these factors in compensation estimation is minimal. As a result, farmers frequently reject compensation payments for their land, as they perceive its monetary value to be lower. Moreover, the compensation provided to evicted farmers is often insufficient or undervalued due to inaccurate property valuations [13,14]. In the case of land valuation in Ethiopia, there is no land market due to the country's land policy, which prohibits farmers from selling their land. Only the government has the authority to sell or lease land to other entities. Consequently, farmers who are subjected to land eviction are provided compensation equal to the average total output of the land in the three years preceding the expropriation, multiplied by 10 (represented by the bid vector of 10). However, this bid vector is determined without considering empirical evidence regarding the evictees' willingness to accept compensation, nor does it align with the “will-buyer will-seller” proposition. While the nation's land policy protects against a buyer-seller dynamic, obtaining an appropriate bid vector by properly evaluating the willingness to accept compensation is difficult. Therefore, estimating the willingness to accept compensation based on empirical evidence is a quest to address the challenges faced by the evictees.

In cases where there is no land market, the contingent valuation technique (CVM)^1^ is the widely applied methods used to estimate WTA^2^ compensation. The various CVM methods are open-ended questionnaires, bidding games, payment card systems, and dichotomous choice models where the first three techniques have their own limitations. For example, the payment card format and bidding games tend to result in lower willingness to accept estimates compared to open-ended formats that affects welfare ness. Hence, both methods of estimation techniques have negative welfare effect on evicted farmers [15]. On the other hand, open-ended questionnaires may suffer from unanswered or inconsistent responses. Therefore, researches such as [9,[16], [17], [18]] employed these methods. However, application of these techniques has methodological gaps in estimating the WTA compensation. In order to fill this methodological gap, ensure consistency and minimize random answers, the this research paper employed the dichotomous choice model to fill the gap using the single-bounded and double-bounded dichotomous choice approaches [19,20]. Therefore, estimating the WTA compensation using appropriate methodological approaches, Dichotomous choice model, can contribute to provide precise robust estimation and valuable insights to the academic knowledge on this subject. Moreover, the study is anticipated making substantial contribution to scientific literature, understanding, and policy input. The empirical findings concerning farmers' willingness to accept compensation can provide valuable intuitions for similar towns across the country, particularly within the region due to the homogeneity of the towns. Furthermore, this with other previous studies can be used as input for future empirical result, and the findings may serve as an updated account for policy changes concerning willingness to accept compensation in peri-urban areas where land eviction is inevitable.

Hence, the objective of this study is to estimate the willingness to accept compensation of peri-urban farmers using contingent valuation method approach in selected towns of Amhara region, Ethiopia.

## 2 Materials and methodology

### 2.1 Description of the study area

The research was conducted in Injibara, Burie, and Gish Abay towns of the Amhara National Regional State. Injibara is the administrative centre of Awi Zone, located 135 km southwest of Bahir Dar and 420 km northwest of Addis Abeba. According to the 2007 census report, the entire population was anticipated to be 21,065; by 2023, it was expected to increase to 56,723 people. It was established in 1991 at a location called Kosober and connects the route from Addis Abeba to Bahir Dar with the road heading west towards the Grand Ethiopian Renaissance Dam.

Established in 1608, the town of Bure is located in western Ethiopia and acts as the administrative center of the district. Situated in the Amhara Region's west Gojjam Zone, it serves as an important trading center and a link between Shewa, Gondar, and Wolega. The coordinates of Bure are 10°42′0″ N 37°4′0″ E, and its elevation is 2091 m above sea level. It is also one of the largest hubs for agricultural and industrial parks in the country.

**Fig. 1:**
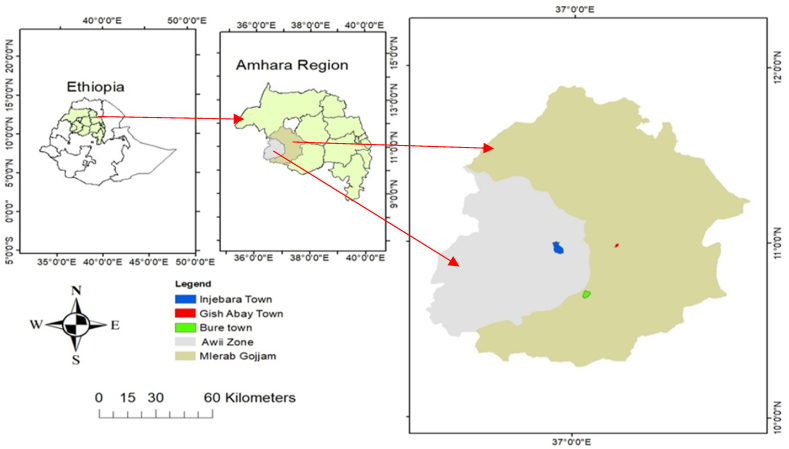
Map of the study areas.

In west-central Ethiopia, in the Amhara Region's West Gojjam Zone, is located the town of Gish Abay. It is the administrative centre of Sekela woreda and is named after Mount Gish and the Abay River (Blue Nile), which has its source on the mountain's slopes. A 39-km gravel road connects the town to Tilili, which is located on the main Addis Ababa-Debre Markos-Bahir Dar Road. At 2744 m above sea level, Gish Abay is located with coordinates of 10°59′N and 37°13′E [21] (see Fig. 1).

### 2.2 Data type, source and data collection tools

To investigate the willingness to accept compensation, both qualitative and quantitative data were collected from respondents. Qualitative data focused on capturing subjective experiences, opinions, and behaviors through descriptive and non-numerical methods, such as household surveys, face-to-face interviews, focus group discussions, and key informant interviews. Quantitative data, on the other hand, was measured and analyzed using statistical techniques. The research employed primary data sources, including surveys and interviews, as well as secondary data sources from relevant literature and institutions. A mixed-methods approach was used, combining both quantitative and qualitative methods to gain a comprehensive understanding of the research problem. Random sampling techniques, including simple random and systematic random sampling, were employed to select representative respondents from the population. The selection process involved where Amhara region was selected purposively because of its fastest growing rate of urbanization compared to other regions and the national average, Awi and West Gojam zones were selected randomly in order to have representativeness of the region, and the three municipalities of Injibara, Burie and Gish Abay were selected purposively because of their fast urbanization rate in region's towns expansion features, and relevance to the study. The final sample size of 393 farm households was determined using [22] formula given by equation (1),

(1) $$n=\frac{z^{2}\times p\times q\times N}{e^{2}\left(N-1\right){+z}^{2}\times p\times q}$$

where n = the number of sample sizes, z = the value of the standard variate (z = 1.96), P = sample proportion, q = 1-p, and e = acceptable error. *N* is the total number of populations.

The sample size of the households is calculated as follows:

$$n=\frac{{\left(1.96\right)}^{2}\times 0.5\times 0.5\times 8060}{{\left(0.07\right)}^{2}\left(8060-1\right){+\left(1.96\right)}^{2}\times 0.5\times 0.5}\approx 393$$

As a result, 393 respondents were randomly chosen from the roasters at each kebele administrative office using the stratified sampling frame that was produced using the systematic random sampling technique. The data was collected from September 2022 to January 2023 and the data was collected from respondent with face-to-face (in person) interview (see Table 1).

**Table: 1:** Distribution of sample households by municipalities.

Municipality	Peri-urban Kebele	Target population	Selected respondents/sample size
Burie	Wan gedam	846	39
	Fereswega	777	36
	Wundigi	543	25
	Derequa mewucha	761	35
Injibara	Besena-basa −01	971	45
	Bahunk Michael- 02	1134	53
	Charate gebriel-03	806	37
	Akaita-04	748	35
	Abo gumbi-05	672	31
Gish Abay	Abay	466	31
	Sangib	340	25
Total		8060	393

#### 2.2.1 Design of the questionnaire and value elicitation format

The contingent valuation method relies on direct responses from individuals, collected through questionnaires, as data sources [23]. In addition to the willingness to accept questions, the survey recommended by the National Oceanic and Atmospheric Administration (NOAA) includes various other inquiries such as income, age, prior community information, curiosity about the community, attitude towards compensation, belief in scenarios, and more [24]. Different response modalities have been utilized in the contingent valuation literature to determine respondents' willingness to accept [25]. The referendum format, known as dichotomous choice, is theoretically the most effective and recommended by the NOAA panel. It has become the preferred elicitation technique for contingent valuation practitioners. Other approaches have demonstrated issues with incentive compatibility, as participants may manipulate results by providing information about their true willingness to accept [26].

The bid vector is a factor used to multiply the output of land taken from evictees, providing cash compensation for the farmers. Selecting appropriate bid prices can enhance the effectiveness of the dichotomous choice parameter and welfare estimations. Pre-testing, either through a survey or focus group discussions, helps determine the bid distribution before the main survey. The survey was conducted among 60 randomly selected households to identify suitable starting bid vectors, ensuring that the questionnaire was both efficient and valid. The data from the pilot survey indicated responses ranging from 10 to 40. Consequently, the starting bid vectors for willingness to accept were set at 10, 20, 30, and 40, with subsequent bid prices determined by doubling and halving the initial amounts.

#### 2.2.2 Theoretical framework of contingent valuation approach

The contingent value approach offers a variety of ways to measure willingness to accept in empirical investigations and research. These choices fall into four main categories: payment cards, open-ended inquiries, dichotomous choices, and bidding games. Single-bounded dichotomous choice and double-bounded dichotomous choice are two sub-methods that fall within the dichotomous choice category [27]. The bidding game has the advantage of being more market-realistic than asking respondents one open-ended question about their maximum willingness to pay because it only requires “yes” or “no” responses to each offer.

The interviewer offers bids to interviewees until the highest willingness to pay is reached. But compared to the open formats, the suggested game provides a much higher average WTP [27,28]. Additionally, the willingness to pay estimates obtained with other closed-ended formats are typically higher than those obtained with the credit card elicitation technique. These days, researchers are less likely to use these two approaches because of the aforementioned drawbacks. People who use the open-ended contingent value approach are asked to indicate how much they would be ready to pay for a good, service, or policy. This approach saves a lot of time because people can readily respond to the questions. Moreover, there is no starting point bias in this method [29]. Numerous investigations, including those by Ref. [9], and [30], have employed the open-ended technique. However, this approach has been criticized for a number of reasons, including the potential for a large number of unanswered inquiries, a lack of interest to provide accurate answers, and offensive responses [31]. [32] developed single-bonded dichotomous choice contingent valuation as a solution to these issues. The interviewee selects a price from a range of bids using this procedure. After then, the bidder is invited to reply with a Yes or No. The similarity of this method with shopping in a real market and the simple process of data collection and easier estimation are among the advantages of this method [33]. Nevertheless, one of the issues with this approach is that it requires a large amount of data in order to estimate the willingness to pay distribution and ascertain the degree of willingness [27]. Accordingly [19,20], introduced the double-bounded dichotomous choice. This technique involves giving the interviewee a second bid that could be greater or lower than the first. To prevent inconsistent results and random responses in the contingent valuation approach, it is appropriate to employ a dichotomous choice questionnaire together with additional questions [34]. Many researches have used dichotomous choice questionnaires to evaluate people's preferences [32,35,36].

From a psychological standpoint, skepticism, ambiguity, and the amount of information at hand affect people's decisions about how much to charge for a product. As such, responses to the second bid on a double-bounded dichotomous choice questionnaire can be impacted by the responses to the first offer [37]. One way to deal with this problem is to create an ideal bid vector and carry out precise surveys and interviews. However, there are some fundamental obstacles in designing a questionnaire, such as choosing the right range and values for the bid vector and allocating the sample size among these values [38]. It is vital to take into account various offer prices in order to fully represent the willingness to accept distribution [39]. Regretfully, a bid vector is not shown in many research; instead, just the first bid is shown. According to Ref. [40], it is insufficient to estimate willingness to accept and the factors that determine it based only on the first bid in such circumstances. Bids are frequently set using pre-questionnaires rather than a scientifically sound methodology, which can result in biases in mean willingness to accept [41].

Various studies have applied the method to obtain optimal bid vectors [16,42]. People frequently struggle to imagine their utility function and are ignorant of their preferences, according to Ref. [33]. As a result, individuals may give truthful answers when questioned about their willingness to accept for a land they have already dispossessed. They are unsure about the price range, nevertheless, when asked if they are willing to accept for the good they have never used before. In fictitious market scenarios, cheap talk scripts have been the subject of numerous research, which have shown that they are an effective means of reducing bias [43].

A. Modelling Framework

The core model for examining dichotomous contingent valuation responses is the random utility model [27,28]. It is assumed that each person's observed discrete choice response in the random utility model represents a utility maximisation process. The status quo, or q^0^, and a particular level of progress, or q^1^, are the two possible degrees of livelihood status involved. Assume for the moment that the representative household receives money in return for the expropriated land. Hence, each household's utility function at status quo (no compensation) is:

(2) *U*_*0i*_ *=* *U(y*_*i*_*, z*_*i*_*, q*^*0*^*, ε*_*0i*_*)*

Individually, household's utility function with compensation is described by equation (3) as follows:

(3) *U*_*1i*_ *=* *U(y*_*i*_*, z*_*i*_*, q*^*1*^*, ε*_*1i*_*)*

By combining equations (1) and (2), we can find equation (4):

(4) *U*_*ji*_ *=* *U*_*j*_*(y*_*i*_*, z*_*i*_*, q*^*i*^*, ε*_*ji*_*)*

Where i = 1,2, …,n indicates individual households; j = 0; 1 symbolizes the two distinct livelihood status; U_0i_ and U_1i_ reflect indirect utilities at the status quo and hypothetical better scenario, respectively; Y_i_ is the highest individual household's discretionary income; z_i_ is independent and design variables (first bid levels, etc.); q_i_ is the quality of the land being estimated; and ε_ji_ is a vector of additional variables that the utility maximizer is aware of, but which are not observed by the researcher and are presumed to have a zero mean.

Note that the household's utility changes from U_0i_ = U (y_i_, z_i_, q^0^, 0_i_) to U_1i_ = U (y_i_, z_i_, q^1^, 1_i_) when the status of the land changes from q^0^ to q^1^ (due to a policy change). Here, q^1^ > q^0^ demonstrates that the suggested scenario or cash compensation has a greater benefit than the current situation. According to economic theories, a rational consumer should try to maximise utility within their means. Income, the presence of remuneration, and other socioeconomic and demographic characteristics are thought to be sources of utility. The given bid is accepted by the household because it increases their income [31]. Individual i therefore responds “yes” to the bid amount ti is assumed by equation (5) as follows:

(5) *(y*_*i*_ *+* *t*_*i*_*, zi, q*^*1*^*, ε*_*1i*_*)* *>* *U (y*_*i*_*, z*_*i*_*, q*^*0*^, *ε*_*0i*_)

We can only infer probabilities from “yes” or “no” responses due to uncertainty to know the random choice. The likelihood that someone will answer “yes” is the likelihood that they believe the proposed scenario is better for them, even with the offered pay, resulting in U_1_ > U_0_. The likelihood that an individual will select utility maximizer i and respond “yes” is provided by equation (6):

(6) *Pr(yes)* *=* *pr(U(y*_*i*_ *+* *t*_*i*_*, z*_*i*_*, q*^*1*^*, ε*_*1i*_*)* *>* *U (y*_*i*_*, z*_*i*_*, q*^*0*^*, ε*_*0i*_*))*

Two modelling decisions are required, per [44], for the parametric estimate of the aforementioned model. We must first decide on a functional form for the expression U(y_i_ + t_i_, z_i_, q^1^, ε_1i_). The error term's distribution must also be stated. Whether they employ a utility differential model or a random WTA model, most applied empirical studies begin by assuming a utility function that is additively separable into systematic and stochastic components of preferences [3,30] which is explained by equation (7):

(7) *U*_*j*_(*y*_*i*_*, z*_*i*_*, ε*_*ji*_) = *V*_*j*_*(y*_*i*_*, z*_*i*_*) + ε*_*ji*_

The likelihood that utility maximizer will provide a favorable answer to the valuation question is as follows, equation (8), given the specification in equation (7):

Pr (yes) = pr (v_1_ (y_i_ + t_i_, z_i_, q^1^) + ε_1i_ > v_0_ (y_i_, z_i_, q^0^) + ε_0i_

(8) = *pr (v*_*1*_*(y*_*i*_ *+* *t*_*i*_*, z*_*i*_*, q*^*1*^*) - v*_*0*_*(y*_*i*_*, z*_*i*_*, q*^*0*^*) + > ε0i* - ε1i

Note that the following is the likelihood that the utility maximizer will respond negatively, rejecting the compensation is explained by equation (9):

(9) *Pr (no)* *=* *1 – pr (yes)*

Although this equation is still too general for parametric estimate, it is possible to assume that the systematic component of the preference function is linear in income and other factors. This means that the equation does not indicate the likelihood of preferring the compensation bid vector in order to decide whether they accept or reject it. Therefore, it has been converted to the reduced form of the equation. Hence, the model can then be reduced to the following equation (10):

(10) *Pr (yes)* *=* *pr (αz*_*i*_*+ βt*_*i*_*+ ε*_*ji*_ *>* *0)*

We suppose that the error term has n_ii_ (0, 1) [26].

Assume that μ = ***ε***_***0i***_ - ***ε***_***1i***_ and F_μ_ ( ) is the cdf of μ, then the probability that the household is willing to accept compensation is given by equation (11):

(11) Pr (yes) = Fμ (ΔV)

Pr (no) = 1 - Fμ (ΔV)

where, *ΔV = (v*_*1*_ *(y*_*i*_ + *βt*_*i*_*, z*_*i*_*, q*^*1*^*) – v*_*0*_ *(y*_*i*_*, z*_*i*_*, q*^*0*^).

Keep in mind that the analysis' primary goal is to determine WTA and generate a WTA function from the presumptive utility function. If Wi represents the household's actual but unobservable WTA for compensation, then it is depicted by equation (12):

*W*_*i*_ = *αz*_*i*_*+ β (y*_*i*_*)*

(12) *α*_*0*_*z*_*i*_ + *β(y*_*i*_ + *ε*_*0i*_*)* *=* *α*_*1*_*z*_*i*_ + *β(y*_*i*_ _*+*_ *ti* + *ε*_*1i*_*)* *=* *α*_*i*_*z*_*i*_ + *β(WTA*_*i*_*)* + *μ*_*i*_

Therefore, the household's WTA can be expressed by equation (13):

(13) $$WTAi=\frac{\alpha zi+\mu i}{\beta }$$

The actual WTA for an individual can be expressed as follows: Wi, the unobservable individual's actual WTA for the improved compensation, has a linear relationship to the original bid ti and the variables is given by equation (14):

(14) *WTA*_*i*_ *=* *1 if WTA*_*i*_ *>* *W*_*i*_*and WTA*_*i*_ *=* *0 if WTA*_*i*_ *<* *W*_*i*_

When using DC-CVM, the ith family is asked whether it would be willing to accept the first offer (ti) in exchange for a specific compensation improvement. The following can be used to show the likelihood of a “yes” or “no” response is given by equation (15):

(15) *pr(yes) to t*_*i*_ *=* *pr(w*_*i*_*)* *≥* *t*_*i*_*and pr(no) to t*_*i*_ *=* *pr(W*_*i*_ *<* *t*_*i*_*).*

With the model's theoretical foundation in place, we can describe the empirical models for DBDC and SBDC in the section that follows.

## 3 Result

### 3.1 Descriptive result

The household heads provided the data. According to the respondents' descriptive results (Table 2), out of all respondents, 63.36 % of households are headed by men, while 36.64 % are headed by women. With a minimum and maximum age value of 21 years and 80 years, respectively, and the average age of the selected respondents is 44.9 years. The selected respondents have 3.2 family members on average, with the minimum and maximum values being 1 and 8 respectively. The respondents' average dependency ratio is 1.14, with minimum and maximum values of 0.3 and 4.3, respectively.

**Table 2:** Descriptive result of explanatory variables.

variable	observation	mean	standard deviation	minimum	maximum
Age	393	38.87	10.9	21	80
Sex	393	0.216	0.41	0	1
Education	393	3.56	3.63	0	12
Family size	393	3.75	1.6	1	8
Dependency ratio	393	0.93	0.88	0.3	4.3
Land size	393	0.79	0.42	0	2
TLU	393	2.11	1.6	3	9
Distance to market	393	2.8	1.66	0.5	6
Total income	393	31228.61	7539.12	19625	96640

The average land size of the respondents is 0.79 ha. The minimum and maximum land size of the respondents is 0 and 2 ha respectively. Moreover, the average tropical livestock unit of the respondents is 2.11 with the minimum and maximum value of 0 and 8.1 respectively. The maximum and minimum distance the respondents walk to the market place is 9 km and 3 km respectively with an average distance of 5.8 km. However, the mean annual income of the respondents is 31228.61 birr with the minimum and maximum annual earnings of 19625 birr and 96640 Birr respectively.

The demographic variables based on gender classification are illustrated in the following chart. There are a total of 308 observations for male-headed households and 85 for female-headed households. According to the descriptive results, the average age of male-headed households is 38.9 years, while the average age of female-headed households is 38.6 years. A statistically significant difference does not exist between these categories, as evidenced by a one-way ANOVA test with an F-value of 0.81 and p-value of 0.7921.

Regarding the education level, male household heads have an average of 3.58 years of education, whereas their female counterparts have an average of 3.48 years. The F-value is 0.74, indicating no statistically significant difference in education level between male and female-headed households, with a p-value of 0.7161.

**Fig. 2:**
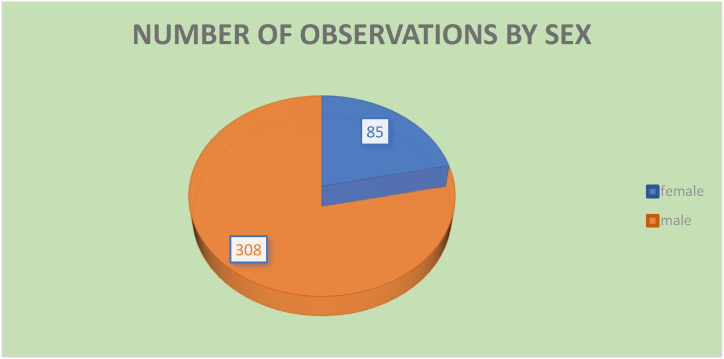
Frequency of the variable sex.

The average family size for male-headed households is 3.7, while for female-headed households, it is 3.95. The ANOVA test yields an F-value of 1.05 with a p-value of 0.3985, suggesting no statistically significant difference in family size between male and female-headed households (see Fig. 2).

Furthermore, the average dependency ratio for male-headed households is 0.936, and for female-headed households, it is 0.939. The F-value is 1.11 with a p-value of 0.3416, indicating there is no statistically significant difference in the dependency ratio between male-headed households and their female counterparts.

**Fig. 3:**
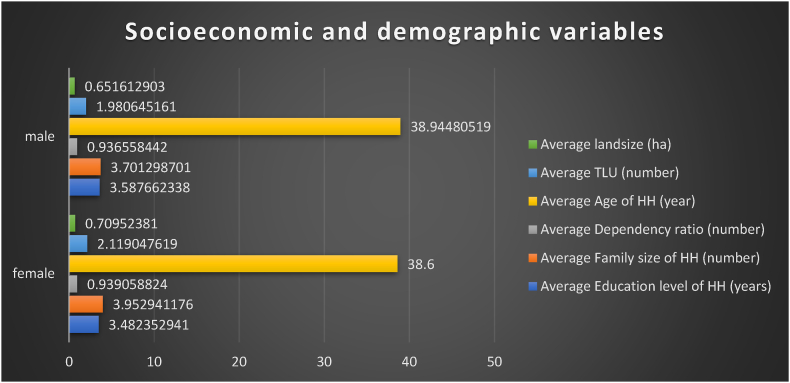
Comparison of the demographic and socio-economic variable by sex category.

The tropical livestock unit (TLU) between male and female headed households is 1.98 and 2.11 respectively. The F-value of the ANNOVA test is 1.63 indicating that there is no statistically significance difference between the two groups. On top of these, the land holding size of the male and female households is 0.65 ha and 0.70 ha respectively. The F-value of the ANNOVA test is 0.63 and p = 7687 depicting there is no statistically significance difference between male-headed households and their female counterparts (see Fig. 3).

### 3.2 Compensation practices

Table 3 presents the trends of the compensation rate in birr. Sample data was taken from displaced households in the towns of Injibara, Burie, and Gish Abay, data on the amount of compensation and land holdings taking expropriations were triangulated with the municipalities of the Land Development and Management department in 2022. According to the information that was made available, 3.349 million Birr^3^ in financial compensation was given for 197 expropriated residents' land holdings totaling 61.9 ha in the aforementioned municipalities.

**Table 3:** The trends of compensation rates in Birr/m^2^/year.

year	Injibara			Burie			Gish Abay			Average compensation
	compensation	Lease price	Allocation price	Compensation price	Lease price	Allocation price	Compensation price	Lease price	Allocation price	
2007	1.52	300	40	1.40	375	30	1.31	–	30	1.457
2008	1.635	950	40	1.601	1950	30	1.30	–	30	1.5454
2009	3.11	1850	40	2.90	2200	40	2.22	–	30	2.833
2010	1.85	3200	50	1.60	3600	50	0.89	–	50	1.255
2011	2.54	4000	50	2.01	4100	50	1.98	–	50	2.1096
2012	1.80	6000	50	1.61	4450	50	1.38	–	50	1.60
2013	2.10	7200	50	1.80	4700	50	1.61	–	50	1.889
2014	2.92	8100	80	2.71	5000	70	2.68	300	70	2.72
2015	2.90	9200	100	2.72	5500	100	2.70	600	90	2.72
2016	2.90	10500	110	2.72	6000	105	2.70	1850	100	2.72
2017	2.92	10900	170	2.71	7200	160	2.69	2200	150	2.72
2018	2.91	11200	210	2.70	9501	210	2.70	3000	200	2.72
Aver	2.1	6116	82.5	1.902	4548	78.75	1.21	1590	75.83	1.674

**Note:** compensation: the compensation price per 1-m square land offered to evicted; lease: the price of 1 m square of land transferred to the third party through the lease system; allocation: the price of land per one square metre collected from housing cooperatives allocated for residence purposes; and average: is the average compensation price of land per 1 m-square paid to evicted of all the study areas.

As a result, the amount of compensation given is 10 times of the average annual output value three years preceding the expropriation. The average nominal land price given as compensation to the evictees was 1.74 birr/m^2^. However, the price varies across municipalities, where Injibara paid 2.1 birr/m^2^, Burie paid 1.902 birr/m^2^, and Gish Abay paid 1.21 birr/m^2^. The compensation also varies across time, where it was given over the periods of 2007–2018. For example, between 2007 and 2018, evictees received an average compensation of 1.74 birr/m2. However, when municipalities leased land to investors, the prices ranged from 300 birr/m2 to 11,200 birr/m2. Additionally, municipalities sold designated land plots for cooperative housing at a price between 30 and 210 birr per square metre. This depicted a significant disparity between the compensation received by evictees and the amount collected by municipalities as compensation from third parties for the same plot of land acquired from the farmers.

### 3.3 Econometric estimation results

#### 3.3.1 Dichotomous model

Table 4 depicts the distribution of bid vectors to each respondent. The distribution of the first bid amount, or t_i_, is one of the first things we want to examine from this data set. Each respondent to the questionnaire, which included dichotomous questions with follow-up, was asked two contingent valuation questions. Table 4 shows the initial bid vector dispersion. Three groups of observations totaling 393 observations each contain a different number of participants.

**Table 4:** Distribution of the bid vectors.

Bid	frequency	percent	cumulative
10	72	18.32	18.32
20	154	39.19	57.51
30	78	19.85	77.35
40	89	22.65	100.00

Table 5 shows the responses to the contingent valuation questions of the bid vectors. When employing contingent valuation data, it's crucial to ensure that respondents are responsive to the bid amount. In other words, we anticipate a higher proportion of respondents to reply positively as the bid amount rises. The result of the model depicted that almost 48.85 % of those interviewed answered yes to the first contingent valuation question.

**Table 5:** Responses to the first contingent valuation questions of the bid vectors.

Answer 1	frequency	percent	Cumulative
No	246	62.60	62.60
Yes	147	37.40	100.00
Total	393	100.00	

#### 3.3.2 Determinants of willingness to accept compensation

The majority of the explanatory variables in single bounded contingent valuation model hypothesised to affect willingness to accept compensation are as expected (Table 6). The estimated regression result depicts that the probability of accepting the offered bid for compensation is positively affected by the bid price and land size of the household at 1 % and 5 % significant levels, respectively. However, the willingness to accept compensation is negatively affected by the age of the household head, the education level of the household head, and the total income of the household at 5 % significance level.

**Table 6:** Single probit regression model.

Dependent variable (Answer 1)	Coefficient	Standard error	z-value
Bid-1	0.1058∗∗∗	0.00836	12.66
Age	−0.0146∗∗	0.0072	−2.02
Sex	0.2264	0.2054	1.10
Education	−0.278∗∗	0.1153	2.41
Family size	−0.0767	0.0666	−1.15
Dependency ratio	0.137	0.12	1.14
Land size	0.3227∗∗	0.0994	3.25
TLU	0.0187	0.0511	0.37
Total income	−0.00003∗∗	0.00001	2.09
Distance to market	−0.031	0.049	0.63
Constant	−1.8644∗∗∗	0.6946	2.68
Observations	393		
Log likelihood	−147.8095		
Prob > chi2	0.000		
LR Chi2(10)	248.99		

(∗∗∗, ∗∗∗, ∗) significant at 1 %, 5 % and 10 % significance level.

The variable bid vector influences the farmers’ decision to accept or reject compensation significantly. The regression coefficient value of the bid vector was 0.1058. This implies that with a one-percent increase in the initial bid vector, the probability of willingness to accept compensation increases by 0.1058 percent, keeping other things constant. The regression coefficient is positive, so it implies that the higher the bid vector, the greater the chance of farmers to accept compensation (WTA). Farmers with a lower initial bid vector often tend to reject the compensation offered. This could be attributed to the signal that a lower initial bid provision implies insufficient compensation, which could potentially jeopardizing their livelihood and place them in a precarious situation [45].

The variable age of the household head negatively influences the farmers’ decision to accept compensation. The regression coefficient value of the age of the household head was −0.0146. This implies that with a one-year increase in the age of the household head, the probability of willingness to accept compensation decreases by 0.0146 percent, keeping other things constant. The regression coefficient is negative, so it implies that the farmers is getting older, the lesser the likelihood to accept compensation (WTA). Farmers with older age often tend to reject the compensation offered. One possible explanation is that elderly farmers may feel that they have a diminishing array of opportunities for starting a new business or finding alternative sources of income. The prospect of finding suitable employment or adjusting to a new lifestyle may seem intimidating and impractical [46].

The variable farmers' education level has a significant effect on the willing to accept compensation. The farmers' education level has a regression coefficient of −0.278. According to the regression coefficient, a one-year increase in educational attainment will result in a 0.2784 decrease in the likelihood of willingness to accept compensation. Therefore, it can be concluded that the higher the farmers' education level, the smaller the chance of the willingness to accept compensation by peri-urban farmers. This is due to the fact that farmers with higher levels of education might be more aware of the actual worth of the land they own and the long-term effects of taking payment that doesn't fairly represent it. They might believe that the compensation being paid is insufficient to reflect the value of the land [45].

Another important variable that influences the willingness to accept compensation is the household's landholding size. As the size of the landholding increases by 1 ha, farmers' willingness to accept compensation increases by 0.3227 when compared to households with less agricultural land. This could be the anticipation that the lump sum amount of compensation granted at one time will allow them to purchase other assets, such as automobiles and buildings, which they believe will yield a higher monthly income than the land itself [47].

The total annual income of the respondents is negatively influencing the households’ willingness to accept compensation, indicating that respondents with higher incomes were less likely to be willing to accept compensation than those with lower incomes. As the total income of the farmer increase by one birr, the probability of willingness to accept compensation declines by 0.00003 citrus paribus. This is because high-income farmers might choose the long-term viability of the income stream over a lump-sum compensation payment. They may regard the land as a stable and reliable source of income, which they are unwilling to abandon.

Similar to the single bounded contingent valuation model, the double bounded contingent valuation estimation result is depicted in Table 7. Among the variables expected to influence the willingness to accept compensation, age of the household head and family size of the household affect positively while total income of the household affects negatively.

**Table 7:** Double bounded bivariate probit regression model.

Dependent variable (Answer 1)	Coefficient	Standard error	z-value
Beta			
Age	4.274∗∗	2.333	1.83
Sex	0.2264	0.2054	1.10
Education	0.905	5378.45	0.00
Family size	7.3∗∗∗	2.066	3.62
Dependency ratio	0.137	0.12	1.14
Land size	0.3227	0.199	1.62
TLU	0.0187	0.0511	0.37
Total income	−0.00003∗∗	0.00001	2.09
Constant	32.504∗∗∗	1.432	22.69
Sigma			
Observations	393		
Log likelihood	−394.55		
Prob > chi2	0.000		
Wald Chi2(10)	248.99		

∗∗∗, ∗∗∗, ∗ significant at 1 %, 5 %, and 10 % significance level.

#### 3.3.3 Estimation of the mean willingness to accept compensation

Table 8 also represents the comparison of alternative estimates of the mean willingness to accept compensation. To find out whether utilizing follow-up responses provide more efficient and robust estimation of willingness to accept compensation or not, this section reports the estimates of the willingness to accept from single-bounded dichotomous choice and double-bounded dichotomous choice models. It is possible to compare the effectiveness of several estimate models in estimating welfare. The primary criterion for evaluating the effectiveness of the models can be found in the precision estimates of welfare measures generated using the “doubleb” command process.

**Table 8:** Comparison of alternative estimates of mean willingness to accept compensation.

Models	Mean WTA	Confidence Interval 95 %		ASL^a^	Range (UB-LB)
		doubleb			
		LB	UB		
SBCV	29.2423	27.8487	30.6358	0.000	2.7871
DBCV	29.2725	27.8777	30.6677	0.000	2.7896

a Achieved Significance Level for testing; Ho: WTA ≤ 0 vs H_1_: WTA>0; LB: Lower Bound; UB: Upper Bound; SBCV: single-bounded contingent valuation; DBCV: double-bounded contingent valuation.

The results demonstrate that, by providing a wider confidence interval level, the double bounded bivariate probit model is significantly more successful than the single bounded model. As a result, the double-bounded dichotomous choice model outperforms the single-bounded dichotomous choice model. Finally, the welfare estimate showed that, due to its wider confidence interval, the double-bounded model's mean willingness to accept compensation (29.27 bid vector) estimates are superior to those of the single bounded probit model. Therefore, we have chosen the estimated result from the double bounded bivariate probit for welfare aggregation in the study area.

### 3.4 Discussion

The main objective of this study is to determine the total willingness to accept compensation for the land that has been taken away from the towns of Injibara, Burie, and Gish Abay in the peri-urban areas due to horizontal urban expansion. Out of the 393 participants surveyed, 197 individuals were evicted and had their possessions confiscated. The total land area seized from them amounted to 619,200 square meters. They were paid a total of 3,349,000 birr, which was calculated as the average annual income of the land three years preceding the expropriation time multiplied by ten. However, the municipalities acquired the land from the farmers at a much lower price compared to the value they paid to the evictees when transferring it to a third party through the lease system. The data obtained from the municipalities in the study areas revealed that the average price of land per square meter transferred to a third party through leasing was 3700 birr, ranging from 300 to 11,200 birr depending on the specific location and municipality. Additionally, the municipalities allocated the land for residential purposes to those in need of constructing houses through housing cooperatives. The municipalities received compensation from these cooperatives for the land taken from the evictees, with an average price of 183.12 birr per square meter, ranging from 30 to 500 birr based on spatial and temporal factors. The data indicated that both in the lease and allocation schemes, there was a significant disparity between the compensation price paid to the evicted farmers and the price at which the land was transferred to the third party. This highlights the considerable impact of the existing compensation price on the livelihoods of the affected individuals. While it is not expected for the average compensation price given to evicted farmers and the lease price established by the municipalities to be equal, minimizing the gap between government revenue and compensation for the evictees can foster a sustainable rural-urban growth relationship. Therefore, mean willingness to accept compensation might theoretically be accomplished without having an impact on the evicted farmers by combining the maximized bid value of compensation with the annual revenue provided by a specific piece of land. Evictees are willing to take compensation for an average bid vector of 29.27, based on the study's findings. This indicates a multiplicity of 29.27 by the average output of the land for the three years prior to the expropriation. Nonetheless, ten times the average output of the land before the expropriation is being paid in compensation to the evicted by the municipalities.

The sampled evictees would have received compensation worth 9,802,523 birr, or roughly 2.9 times more, than what they actually received. The descriptive finding that evictees (as compensation) and municipalities (via lease and allocation schemes) got larger disparities in revenue generation from the same piece of land. This result conforms with that of [48]. The evictees are not happy with the government's current ten-year bid vector because of this major undervaluation of the land's compensation price, even though the compensation they have received so far is commensurate with what should be paid.

## 4 Conclusion and recommendation

Based on the results and discussion, it can be inferred that in the peri-urban areas of Burie, Injibara, and Gish Abay towns, the willingness-to-accept (WTA) compensation among farmers stood at a bid vector of 29.27. Key determinants affecting farmers' decisions on accepting or rejecting compensation included the initial bid vector, age of the household head, household land size, education level of the household head, and annual income of the household. The displacement of farmers from their land as a result of urban expansion driven by development is unavoidable, but it should be approached in a manner that benefits both parties, ensuring that indigenous farmers are not adversely affected. This goal can be attained by utilizing empirical evidence to examine the willingness-to-accept (WTA) compensation of peri-urban farmers. Consequently, it is crucial to provide appropriate compensation to those who are displaced, and ongoing support should be provided to assist them in sustaining their livelihoods post-expropriation. This is essential because these individuals may lack the financial and administrative skills necessary to effectively manage a lump sum payment if it is offered all at once. The broader implication of addressing these issues is vital improving social justice, inform sustainable economic development, conflict mitigation, and community well-being. Therefore, the government ought to embrace multifaceted strategies in handling land valuation issues to guarantee fairness, transparency, and efficiency. Here are some policy suggestions that the government should contemplate implementing:

• Informed Property Valuation: Professional judgments and data-driven methodologies could be utilized to precisely evaluate property values. This can help to avoid undervaluation or overvaluation of properties during transactions.
• Informed Property Valuation: Utilizing expert opinions and data-driven methods to accurately assess the value of properties. This can help in preventing undervaluation or overvaluation of properties during transactions.
• Planned Expropriation: When the government needs to take private property for public objectives, a well-defined and fair expropriation procedure can assist safeguard property owners' rights.
• Comprehensive Compensation Calculations: Ensuring that when the government expropriates the land for public purpose, the owner receives appropriate and fair compensation. This entails taking into account a number of variables, including the property's market value, any upgrades performed, and any inconveniences experienced by the owner.
• Awareness Campaigns: Educating the public on property rights, valuation processes, and legal aspects of land transactions to empower landholders and encourage willingness in the industry.
• Early Provision of Residential Homes: To reduce life disruptions, it is important to make sure that communities or individuals impacted by land eviction have access to appropriate alternative housing options as soon as possible.
• Shareholdings: Investigating options such as allowing land owners to have a stake in development projects in cases of expropriation or development, thereby enabling them to benefit from the future value of the property.
• Accountable Valuation: Implementing mechanisms to hold property valuation professionals and institutions accountable for their assessments, ensuring accuracy and integrity in the valuation process.
• Property Valuation Institutions: Establishing separate institutions dedicated to property valuation that adhere to best practices and standards, thus enhancing the credibility and reliability of valuation processes.

Finally, subsidizing evicted farmers can help them sustain their livelihoods, taking inspiration from the compensation subsidy model in Kenya. Eviction often affects farmers' economic activity and can result in income loss. Governments may protect evicted farmers' livelihoods while supporting long-term growth and resilience in peri-urban communities and adjusting assistance programs to local situations. Subsidizing evicted farmers is a reasonable and compassionate way to support individuals suffering displacement. This can help farmers fill financial gaps and avoid severe economic shocks. This assistance can serve as a safety net during transitional periods, backup farmers to navigate uncertainty and sustain their livelihoods in the face of eviction. Abrevaitions are depicted in Table 9.

**Table 9:** Abbreviations.

Abbreviation	Description
DBCV	Double-Bounded Contingent Valuation
SBCV	Single-Bounded Contingent Valuation
OECD	Organization of Economic Cooperation for Development
NOAA	National Oceanic and Atmospheric Administration
WTA	willingness to Accept
WTP	Willingness to Pay

## 5 Area for future research

This study employed three towns, Injibara, Burie, and Gish Abay, as case studies to analyze farmers' willingness to accept compensation after being evicted from their land. However, the long-term economic, social, and psychological impact of compensation acceptance on evicted farmers, the community's post-expropriation resilience to prevent vulnerabilities, and the gender dynamics influencing compensation acceptance (i.e., how compensation affects men and women differently) are all important considerations. By delving deeper into these subjects for future research, scholars can contribute to a better understanding of the complexities surrounding evicted farmers' willingness to accept compensation, as well as provide more informed policy decisions and actions in this area.

## CRediT authorship contribution statement

**Tadele Alamneh:** Writing – original draft, Software, Methodology, Investigation, Formal analysis, Data curation, Conceptualization. **Melkamu Mada:** Writing – review & editing, Validation, Supervision, Conceptualization. **Tora Abebe:** Writing – review & editing, Supervision, Conceptualization.

## Informed consent

Verbal agreement was acquired from participants voluntarily and openly prior to the data collection procedures of this study. The authors explained to the participants that the collected data were merely utilized for academic reasons and that no fees were offered to the research subjects (participants). The authors also explained verbally to the study participants how participant anonymity was safeguarded, how data collection was done, the ability to request questions, the freedom to revoke consent at any time, the lack of penalty for doing so and the research benefits. Finally, the participants verbally granted the authors informed consent. Individual interviews and focus groups were conducted in Amharic (the main local language spoken in the study area and the official working language) to address their demands.

## Ethical consideration

Ethics are the moral principles that direct the actions of an individual or a group of people. To commence the research activities, the authors have received ethical letters from Arba Minch University School of Graduate Studies of Doctoral Programs Coordination Office (with ID-DPc/175/15/25/01/2016). From the beginning up to the end of our work, the authors of this manuscript have strived for academic honesty and overcame the tendency to discount data that did not match our requirements. We respected the activities, the norms, and morals of all the research units as well as others. In addition, potential conflicts of interest, plagiarism, proper authorship credit, and ethical study data management and disclosure were all considered by the authors of this manuscript. The research participants were duly informed about the objectives and advantages of the study, and their consent was required voluntarily. Generally, participants in the study were treated equally regardless of their gender, age or race, and did not incur any unwarranted risks. Participants were provided with all necessary information to make their own decision on whether or not to participate in the study. So, the authors believed that the ethical principles that we followed in this manuscript guaranteed that the rights and welfare of individuals were protected.

## Data availability

Data will be made available from the corresponding author up on reasonable request.

## Funding

This research did not receive any specific grant from funding agencies in the public, commercial, or not-for-profit sectors.

## Declaration of competing interest

The authors declare that they have no known competing financial interests or personal relationships that could have appeared to influence the work reported in this paper.

## Figures and Tables

**Table 1:** Percentage of urban population in 2007 and projected percentages, 2017-37.

Region	% of urban population			Rate of urbanization in %	
	Census	projection			
	2007	2017	2037	From 1994 to 2007	Projected 2007–2037
Amhara	12.3	17.0	37.1	3.94	6.02
Afar	13.4	19.2	50.5	6.62	7.53
Tigray	19.5	26.1	50.5	4.52	5.17
Oromia	12.4	15.0	31.8	4.13	5.12
SNNP	10.3	14.0	24.6	6.04	4.77
Benishangul	14.6	21.2	43.4	7.69	5.94
Gambela	25.4	32.1	60.4	8.03	4.75
Somali	14.0	14.4	18.6	2.71	2.65
Harari	54.2	55.7	62.4	2.02	2.30
Dire Dawa	67.9	69.3	74.8	2.28	2.20
Addis Ababa	100	100	100	2.10	2.10
National average	16.1	20.4	31.1	3.77	3.86

## Footnotes

1 **CVM** is a technique used in economics to determine the economic value that individuals place on a non-marketable good or service.

2 **WTA** refers to the minimal amount of compensation that an individual is willing to receive in exchange for giving up or foregoing a good.

3 Birr is the currency of Ethiopia where the average exchange rate of one birr = 0.0193 US Dollar in 2022.
